# Dihydrotanshinone I Alleviates Crystalline Silica-Induced Pulmonary Inflammation by Regulation of the Th Immune Response and Inhibition of STAT1/STAT3

**DOI:** 10.1155/2019/3427053

**Published:** 2019-07-09

**Authors:** Yiting Zhang, Chao Li, Siyi Li, Yiping Lu, Sitong Du, Xinning Zeng, Xi Chen, Jie Chen

**Affiliations:** ^1^Division of Pneumoconiosis, School of Public Health, China Medical University, Shenyang, China; ^2^Biobank, The Affiliated Shengjing Hospital, China Medical University, Shenyang, China

## Abstract

Occupational exposure to crystalline silica (CS) results in a persistent pulmonary inflammatory response that eventually leads to abnormal tissue repair, disability, and death. The inflammatory-immune responses occur in the early stages of CS exposure, and both innate and adaptive immunity are involved. CD4+ T cells play a pivotal role in the pathogenesis of CS-induced pulmonary disease, which has no proven curative therapy. Dihydrotanshinone I (DHI), a natural product isolated from Salvia miltiorrhiza Bunge (Danshen), has anti-inflammatory and immunomodulatory properties. However, whether DHI has a protective effect on CS-induced lung disease, how it influences the Th immune response, and the potential underlying molecular mechanism(s) have not been fully clarified. In this study, DHI treatment of CS-exposed mice reduced the expression of proinflammatory cytokines and the infiltration of immune cells. It significantly ameliorated CS-induced pulmonary inflammation by attenuating T helper (Th)1 and Th17 responses, which were tightly related to the inhibition of STAT1 and STAT3. DHI significantly altered Th2 cytokines but not the Th2 nuclear transcription factor. Furthermore, our study found that DHI treatment also affected regulatory T cell activity in CS-injured mice. Taken together, our findings indicated that DHI could modulate Th responses and alleviate CS-induced pulmonary inflammation, suggesting a novel application of DHI in CS-induced pulmonary disease.

## 1. Introduction

Environmental and occupational exposure to crystalline silica (CS) particles represents an unresolved major public health problem affecting large populations on a global scale. Tens of millions of workers worldwide are estimated to be exposed to CS particles [1]. Long-term inhalation of CS particles causes an occupational lung disease, which is especially prevalent in developing countries [2]. This disease is characterized by persistent lung inflammation and aberrant tissue repair [3]. CS-induced pulmonary inflammation begins with the activation of the innate immune response, which produces an environment rich in proinflammatory cytokines and chemokines that induce the infiltration of different inflammatory cells, including neutrophils, macrophages, and lymphocytes [4, 5]. Because CS particles deposited into the lungs cannot be removed, CS-induced pulmonary inflammation persists and leads to the activation of the adaptive immune response, severe pulmonary injury, and ultimately disability and death [6]. Thus, prevention of CS-induced lung disease remains a major clinical challenge in modern society.

CD4+ T cells (e.g., T-helper (Th)1, Th2, Th17, and regulatory T (Treg) cells) play a vital role in coordinating the adaptive immune response and participate in the pathogenesis of the pulmonary inflammation caused by CS exposure [7, 8]. In addition, there is a transition between the Th1 and Th2 responses during the progress of CS-induced lung disease. The Th1 cell response is primarily involved in the early inflammatory stage, whereas the Th2 cell response is associated with late tissue repair [9–11]. Th17 cells, which secrete IL-17A, can recruit inflammatory cells and produce tremendous levels of proinflammatory cytokines that promote lung inflammation [12–14]. Tregs, which are Th response regulators, play a pivotal role in maintaining immune homeostasis and are also involved in CS-induced lung inflammation [15, 16]. Moreover, the networks of cytokines and transcription factors could determine CD4+ T cell fates and produce effector cytokines [17]. Furthermore, recent evidence suggests that the cytokine-activated signal transducer and activator of transcription (STAT) proteins are critical for regulating Th cell differentiation [17–19]. IFN-*γ*-activated STAT1 can induce T-bet expression, which promotes the differentiation of Th1 cells [20, 21] whereas IL-6-mediated STAT3 is important for Th17 differentiation [22]. Therefore, the inhibition of cytokine signaling and STATs could regulate the Th immune response and presents a potential therapeutic approach to alleviate CS-induced pulmonary inflammation.

Although extensive studies have been conducted for decades, the complex pathology caused by CS has not been fully clarified, and there are no curative treatments for CS-induced lung disease [1]. Many anti-inflammatory drugs (e.g., corticosteroids) have limited efficacy and cause adverse effects [23]. Therefore, there is an urgent need to develop new and effective therapies against this disease. Recently, traditional Chinese medicine (TCM) has received increasing attention for treating many inflammatory diseases due to its many biological activities and low toxicities [24, 25]. Dihydrotanshinone I (DHI), derived from the rhizome of Salvia miltiorrhiza Bunge (Danshen), is a diterpene compound with a high medicinal value [26]. Numerous studies have reported that DHI displays a wide range of biological effects, including anti-inflammatory [27–29], antioxidant [30], antibacterial [31], antitumor [32, 33], cardiovascular protection [34, 35], and liver protection [36]. DHI may play a therapeutic role in a variety of inflammatory diseases, including atherosclerosis [37], ulcerative colitis [38], and allergic inflammation [39]. In addition, DHI can modulate immune cell function, including inhibition of mast cell degranulation [39] and suppression of the release of cytokines from immune cells [29, 40, 41]. To date, there have been no studies on the effects of DHI on CS-induced pulmonary inflammation and the Th immune response. In the present study, we examined whether DHI had protective effects against the CS-induced inflammatory response and investigated the potential underlying mechanisms.

## 2. Materials and Methods

### 2.1. Crystalline Silica

Crystalline silica (CS) particles (size distribution: 97% < 5 *μ*m in diameter, 80% < 3 *μ*m in diameter, and median diameter of 1.4 *μ*m) were purchased from the U.S. Silica Company (Frederick, MD, USA). Silica particles were ground in saline for 3 h, boiled in 1 N HCl to remove endotoxins, and suspended in sterile saline. Suspensions were autoclaved and then sonicated for 10 min before use.

### 2.2. Dihydrotanshinone I

**Figure 1:**
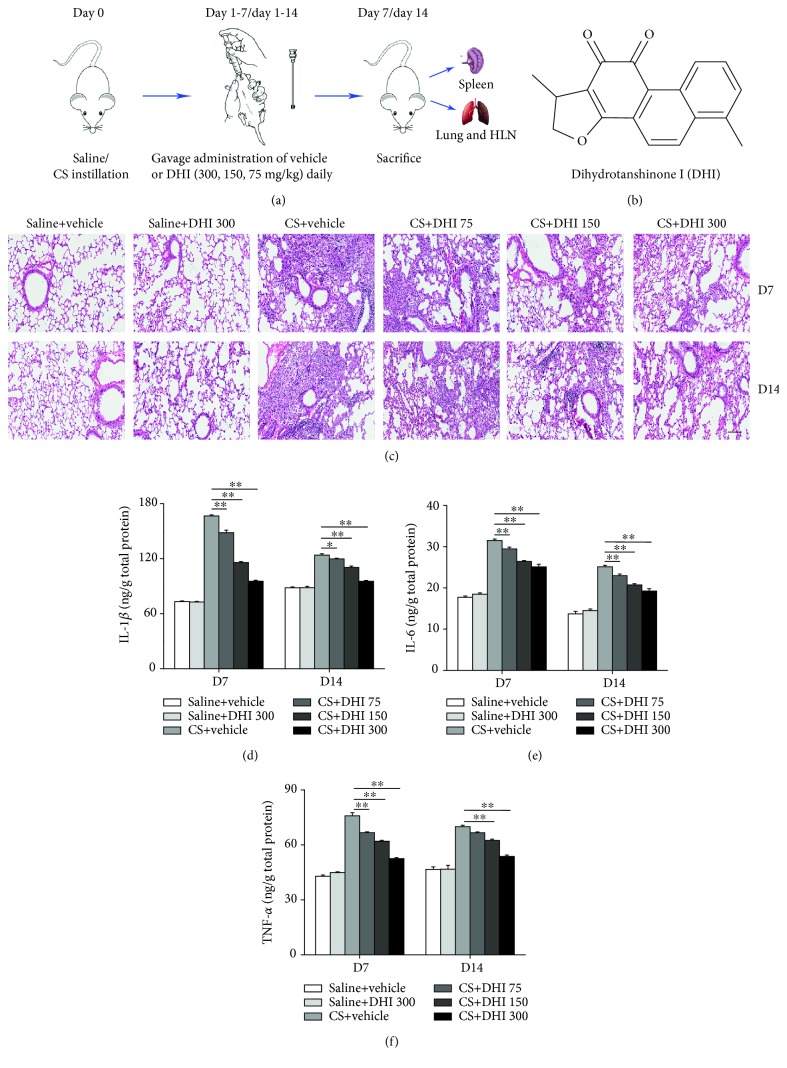
DHI ameliorated CS-induced pulmonary inflammation. (a) Dosage regimen of DHI in an experimental CS-exposed mouse model. (b) The chemical structure of DHI. (c) Effects of DHI on H&E staining of mouse lungs at day 7 and day 14. The scale bar indicates 100 *μ*m (*n* = 3 to 4 per group). (d–f) ELISA analysis of IL-1*β*, IL-6, and TNF-*α* in lung homogenates (*n* = 5 per group). Data are presented as the mean ± SEM. ^∗^*P* < 0.05; ^∗∗^*P* < 0.01. All experiments were performed in triplicate.

Dihydrotanshinone I (DHI, Figure 1(b)) was purchased from Nanjing Plant Origin Biological Technology Co. Ltd. with a purity > 98% as determined by high-performance liquid chromatography. For the animal experiments, DHI was dissolved in 0.5% sodium carboxyl methyl cellulose (CMC-Na; Sigma-Aldrich, St. Louis, MO, USA) solution in distilled water.

### 2.3. Animals and Treatment

Female C57BL/6 mice (6 to 8 weeks) were obtained from SLAC Laboratory Animal Co. Ltd. (Shanghai, China) and housed in a specific pathogen-free facility. The mice were fed a standard mouse feed and water ad libitum. All animals were acclimatized for one week before the start of the experiments. All animal experiments and procedures were performed in strict accordance with the Animal Care and Use Committee at China Medical University, which abides by the National Institutes of Health Guide for the Care and Use of Laboratory Animals.

The mice were randomly divided into six groups based on body weight (10 mice per group), as follows: animals in the Saline group and Crystalline silica group were treated with vehicle (0.5% CMC-Na); in the Saline+DHI group were treated with 300 mg/kg of DHI; and in the Crystalline silica+DHI groups were treated with 300, 150, or 75 mg/kg of DHI, respectively. DHI and vehicle (0.5% CMC-Na) were administered intragastrically (i.g.) once daily for 14 consecutive days. The experimental CS-exposed mouse model was induced as previously described [5]. Briefly, C57BL/6 mice were instilled intratracheally with 50 *μ*L solution containing 2.5 mg CS after anesthetization. Sterile saline was used as a control in the Saline group. As shown in Figure 1(a), mice were sacrifice on days 7 and 14 after intratracheal instillation under anesthesia. Lung and spleen tissues were obtained for further analyses.

### 2.4. Histological Analysis

Lung samples were fixed in 4% paraformaldehyde, embedded in paraffin, and cut into 5 *μ*m sections. The sections were stained with hematoxylin and eosin (H&E) to assess the degree of inflammation. H&E staining was visualized using Leica Aperio CS2 (Leica Biosystems, USA).

### 2.5. Cytokine Analysis by Enzyme-Linked Immunoassay (ELISA)

Cytokines in the BALF (IFN-*γ*, IL-4, IL-13, and IL-17) and lung homogenates (TNF-*α*, IL-6, and IL-1*β*) were measured using commercial ELISA kits based on the manufacturer's instructions. Lung homogenates were standardized to a concentration of 1 *μ*g/*μ*L before testing. All ELISA kits were purchased from R&D Systems (Minneapolis, MN, USA).

### 2.6. Bronchoalveolar Lavage and Differential Cell Counts

Bronchoalveolar lavage fluid (BALF) was obtained by cannulating the trachea and perfusing 1 mL of sterile saline into the lung tissue three times. The BALF was centrifuged at 1500 rpm at 4°C for 8 min, and the supernatants were stored for subsequent cytokine detection. After lysis of red blood cells (RBCs), the BALF cell pellet was washed and resuspended in phosphate-buffered saline (PBS). Total cell counts were determined using standard hematological procedures. After preparation of the cytospin smear, the cells from the BALF were stained using the Wright-Giemsa method to identify macrophages, neutrophils, and lymphocytes in fields of 200 cells using standard morphologic criteria.

### 2.7. Immunofluorescence

Paraffin-embedded lung tissue was cut into 5 *μ*m sections. After deparaffinization and rehydration, microwave antigen retrieval was performed by microwave heating for 20 min in citrate buffer (pH = 6.0). The sections were blocked with 5% BSA for 1 h at room temperature. The samples were then incubated with B220 primary antibody (eBioscience, 1 : 100) overnight at 4°C followed by incubation with Alexa Fluor 594-conjugated secondary antibody (1 : 200). Nuclei were stained with DAPI. Images were captured using a fluorescence microscope.

### 2.8. Isolation of Hilar Lymph Nodes and Spleen Cells

To obtain single-cell suspensions from the hilar lymph nodes (HLN) of mice, HLN were dissected with needles into small pieces and digested using 0.25% trypsin for 5 min at 37°C followed by termination with PBS containing 3% fetal bovine serum. Samples were centrifuged at 1500 rpm and 4°C for 8 min. The HLN cells were washed and resuspended in PBS. Spleens were mechanically dissociated in cold PBS. RBCs were lysed with red blood cell lysis buffer, and splenocytes were washed and resuspended in PBS.

### 2.9. Flow Cytometry Analysis

HLN cells and splenocytes were stimulated with leukocyte activation cocktail (BD Pharmingen, San Jose, CA, USA). The cells were incubated with purified rat anti-mouse CD16/32 (BD Pharmingen) to block the Fc receptors (FcRs) and then stained with primary antibodies (BD Pharmingen) at 4°C for 30 min. Fluorescence-activated cell sorting (FACS) was performed using a BD Accuri™ C6 Plus (BD Bioscience, San Jose, CA, USA). Th1 cells were identified as CD4 and IFN-*γ* double-positive cells. Th17 cells were identified as CD4 and IL-17A double-positive cells. CD4 and forkhead box P3 (Foxp3) were used to identify Treg cells. FACS data were analyzed using FlowJo V10 software.

### 2.10. Quantitative Real-Time PCR (qRT-PCR)

Total RNA was extracted from the lung and spleen using a TRIzol reagent (Life Technologies, USA). cDNA was prepared using the PrimeScript RT kit (Takara, Japan) according to the manufacturer's protocol. qRT-PCR was performed using the SYBR Green Master Mix Kit (Takara, Japan) and ABI 7500 Fast Real-Time PCR System (Applied Biosystems, Foster City, CA, USA). The primer sequences are presented in Table 1. Relative quantification was performed by the 2^–*ΔΔ*CT^ method using *gapdh* as the reference gene.

### 2.11. Western Blot Analysis

Total protein was isolated from the lung tissue cleared of blood using RIPA buffer (Beyotime) containing protease (Beyotime) and phosphatase (Roche, Basel, Switzerland) inhibitors. The Pierce BCA Protein Assay Kit (Thermo Scientific, USA) was used to estimate the protein concentration. Lysates were adjusted to a concentration of 3 *μ*g/*μ*L. The protein samples were separated on sodium dodecyl sulfate-polyacrylamide gels and transferred to PVDF membranes (Millipore, Germany). The membranes were blocked with 5% skim milk for 1 h at room temperature in TBST buffer and probed with the following primary antibodies: anti-STAT1, anti-phospho-STAT1, anti-STAT3, anti-phospho-STAT3 (Affinity, 1 : 500), and anti-*β*-actin (CST, 1 : 1000) overnight at 4°C. After washing with TBST, the membranes were incubated with horseradish peroxidase-conjugated secondary antibody (CST, 1 : 2000). Protein bands were visualized using enhanced chemiluminescence (ECL). *β*-Actin was used as a loading control. ImageJ software was used to analyze the optical density of the protein bands.

### 2.12. Statistical Analysis

All the data were analyzed using SPSS software, version 21.0. Data are presented as the mean ± standard error of the mean (SEM). One-way analysis of variance (ANOVA) followed by the Student-Newman-Keuls test was performed to compare the differences between multiple groups. A *P* value of less than 0.05 was considered statistically significant.

## 3. Results

### 3.1. DHI Alleviated CS-Induced Pulmonary Inflammation

To determine whether DHI could ameliorate CS-induced pulmonary inflammation, we administered different doses of DHI to CS-exposed mice (Figure 1(a)). As shown in Figure 1(c), the CS-exposed mice treated with vehicle had remarkable inflammatory cell infiltration into the lungs compared to saline-instilled mice at days 7 and 14. DHI alleviated this infiltration in a dose-dependent manner. Considering that the accumulated inflammatory cells in the lungs could release large amounts of inflammatory cytokines, we measured the levels of three representative proinflammatory cytokines (IL-1*β*, IL-6, and TNF-*α*) in lung homogenates. As shown in Figures 1(d)–1(f), Expression levels of these cytokines were higher in the lungs of CS-exposed mice compared to those from saline-instilled mice at days 7 and 14. These CS-induced expression levels were notably decreased in a dose-dependent manner by DHI treatment.

**Figure 2:**
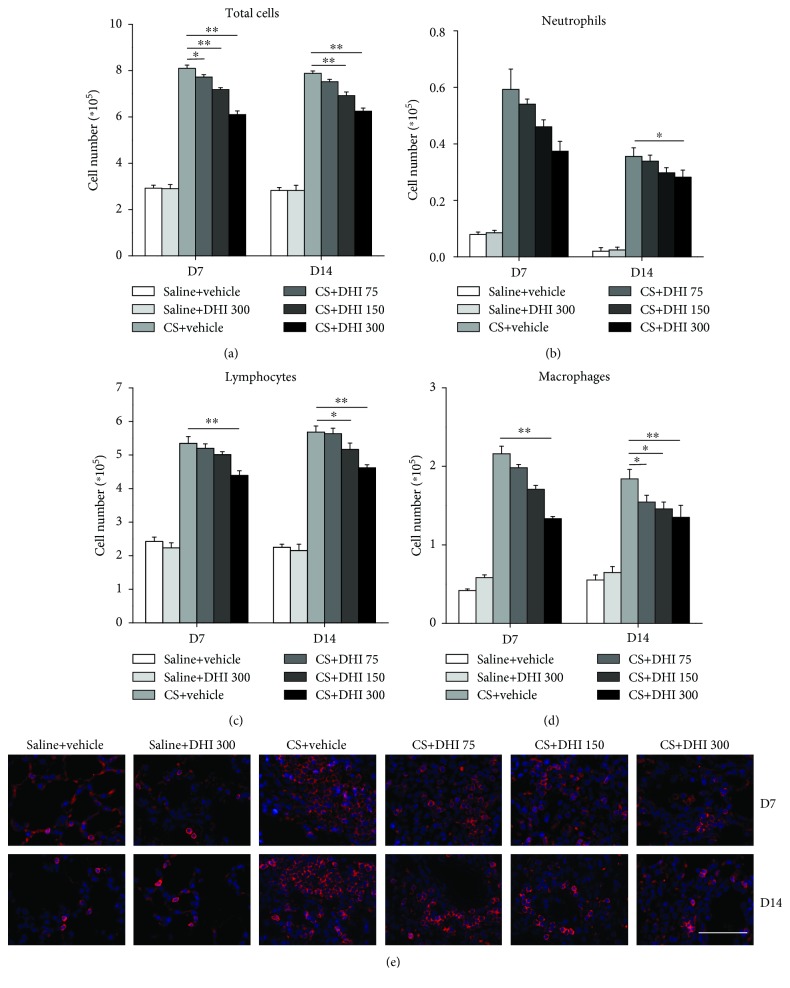
DHI reduced the accumulation of immune cells in a mouse model of CS-induced pulmonary inflammation. (a–d) Cells were stained using the Wright-Giemsa method to identify total cells, neutrophils, lymphocytes, and macrophages in the BALF (*n* = 5 per group). (e) Immunofluorescence analysis of B220-positive B cells in lung sections (red). The scale bar indicates 50 *μ*m (*n* = 3 to 4 per group). Data are presented as the mean ± SEM. ^∗^*P* < 0.05; ^∗∗^*P* < 0.01. All experiments were performed in triplicate.

To better understand the role of DHI in the pathogenesis of CS-induced pulmonary inflammation, we determined the numbers of total cells, neutrophils, lymphocytes, and macrophages in BALF. The total number of cells in the BALF was significantly reduced by DHI compared to that of the CS-treated group (Figure 2(a)). The accumulated CS particles caused a marked accumulation of neutrophils in the BALF at days 7 and 14. In contrast, DHI treatment significantly decreased the number of these cells in the BALF of CS-exposed mice at day 14 (Figure 2(b)). The numbers of lymphocytes and macrophages also significantly declined at days 7 and 14 in the BALF of CS-exposed mice following DHI treatment (Figures 2(c) and 2(d)). Furthermore, the number of B220-positive B cells in the lungs of CS-exposed mice at days 7 and 14 was reduced by DHI in a dose-dependent manner. (Figure 2(e)). These results suggested that DHI could suppress macrophage and lymphocyte infiltration, thus reducing the degree of CS-induced pulmonary inflammation.

### 3.2. DHI Attenuated the Th1 Immune Response in CS-Treated Mice

**Figure 3:**
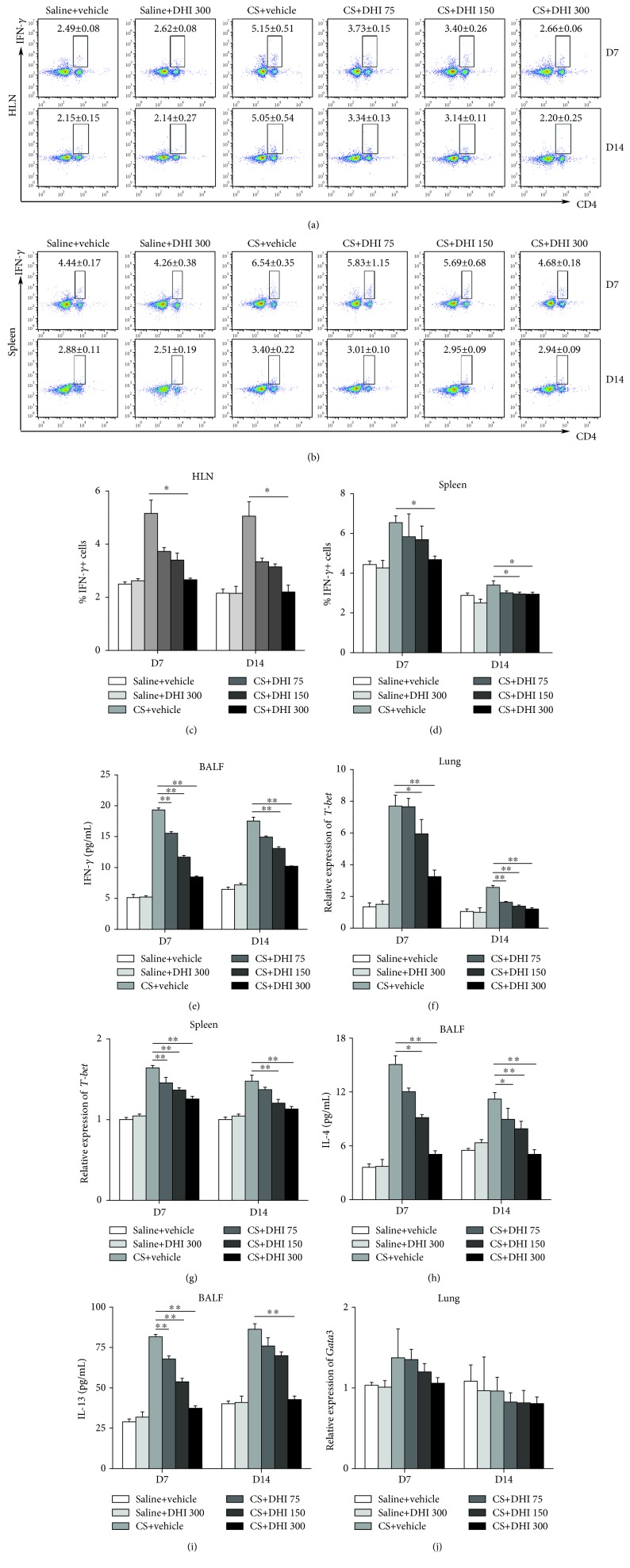
DHI modulated Th1/Th2 immune responses in a mouse model of CS-induced inflammation. Percentage of CD4+IFN-*γ*+ Th1 cells in the HLN (a, c) and spleen (b, d) was detected by flow cytometry (*n* = 5 to 6 per group). (e) ELISA analysis of IFN-*γ* levels in the BALF (*n* = 5 per group). (f, g) Th1 nuclear transcription factor T-bet expression levels in mouse lung and spleen were assayed by qRT-PCR (*n* = 5 to 6 per group). (h, i) ELISA analysis of IL-4 and IL-13 levels in the BALF (*n* = 5 per group). (j) Th2 nuclear transcription factor GATA3 expression levels in mouse lung were assayed by qRT-PCR (*n* = 5 to 6 per group). Data are presented as the mean ± SEM. ^∗^*P* < 0.05; ^∗∗^*P* < 0.01. All experiments were performed in triplicate.

The Th immune response plays a crucial role in the inflammatory pathogenesis caused by CS [42]. Our previous studies demonstrated that the Th1 and Th2 immune responses are involved in the progression of CS-induced lung disease [5]. In the present study, we investigated the effects of DHI on the Th1/Th2 immune response in CS-treated mice. We found that DHI treatment reduced the proportion of Th1 cells in the HLN and spleen of CS-treated mice in a dose-dependent manner at both days 7 and 14 (Figures 3(a)–3(d)). Consistent with this finding, the expression of the Th1 typical effecter cytokine IFN-*γ* in the BALF was also significantly decreased by DHI treatment (Figure 3(e)). Furthermore, an attenuated Th1 immune response was indicated by decreased expression of the Th1 nuclear transcription factor T-bet in the lung and spleen (Figures 3(f) and 3(g)). Interestingly, the levels of the Th2 cytokines IL-4 and IL-13 in the BALF were abrogated by DHI treatment (Figures 3(h) and 3(i)), whereas the expression of the Th2 nuclear transcription factor GATA3 in the lung tissue was not altered by DHI treatment (Figure 3(j)). Collectively, these results showed that DHI treatment could effectively ameliorate the Th1 immune response in the CS-induced inflammation stage.

### 3.3. DHI Reduced the Th17 Immune Response in CS-Treated Mice

**Figure 4:**
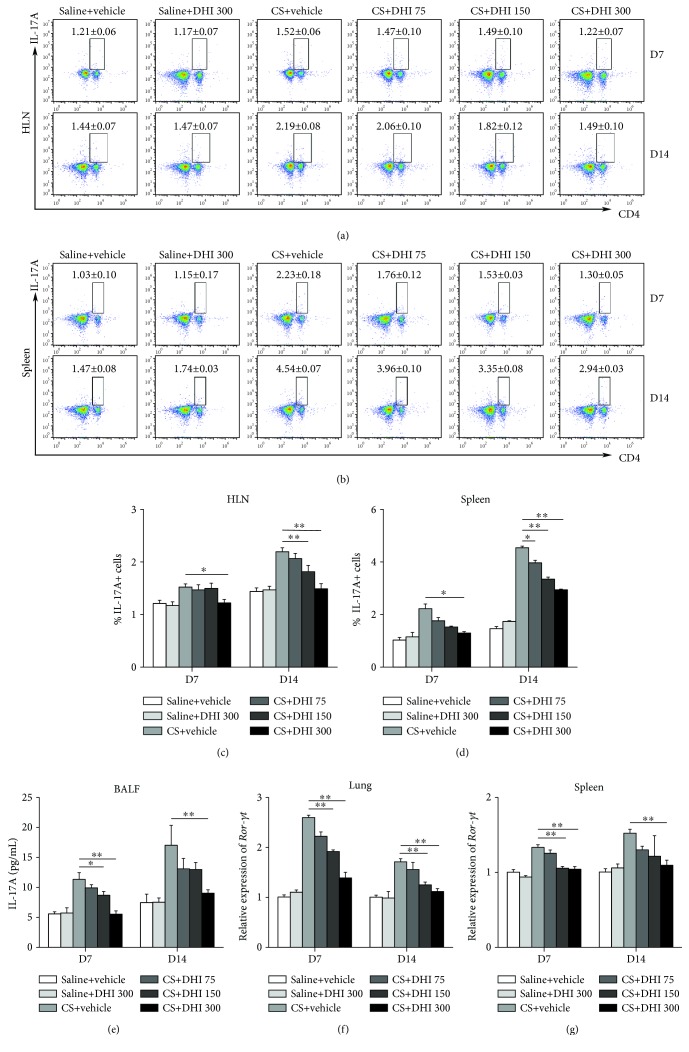
DHI modulated the Th17 immune response in a mouse model of CS-induced inflammation. The percentage of CD4+IL-17A+ Th17 cells in the HLN (a, c) and spleen (b, d) was detected by flow cytometry (*n* = 5 to 6 per group). (e) ELISA analysis of IL-17A levels in the BALF (*n* = 5 per group). (f, g) Th17 nuclear transcription factor ROR-*γ*t expression levels in mouse lung and spleen were assayed by qRT-PCR (*n* = 5 to 6 per group). Data are presented as the mean ± SEM. ^∗^*P* < 0.05; ^∗∗^*P* < 0.01. All experiments were performed in triplicate.

Previous studies have shown that Th17 cells and IL-17A play important roles in CS-induced pulmonary inflammation [43, 44]. In the present study, the percentage of Th17 cells (CD4+IL-17A+) was significantly enhanced in the HLN and spleen of CS-injured mice compared to those of saline-treated mice; this effect was reversed by DHI treatment (Figures 4(a)–4(d)). IL-17A levels in the BALF were also reduced (Figure 4(e)). In addition, the expression levels of the Th17 nuclear transcription factor ROR-*γ*t were decreased following DHI treatment (Figures 4(f) and 4(g)). These findings suggested that the Th17 immune response was alleviated by DHI treatment at the inflammatory stage of silicosis.

### 3.4. Regulatory T Cells Were Influenced by DHI Treatment

**Figure 5:**
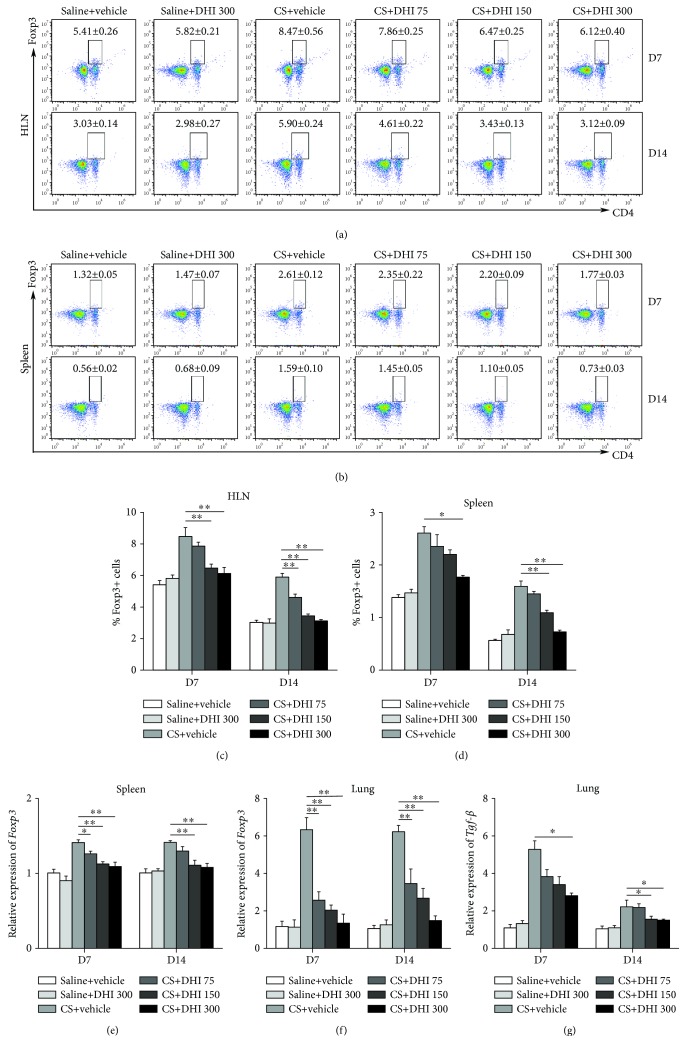
DHI regulated T regulatory cells in a mouse model of CS-induced inflammation. Percentage of CD4+Foxp3+ T regulatory cells in the HLN (a, c) and spleen (b, d) was detected by flow cytometry (*n* = 5 to 6 per group). (e, f) Foxp3 mRNA level in the spleen and lung was assayed by qRT-PCR (*n* = 5 to 6 per group). (g) TGF-*β* mRNA level in lung tissues was assayed by qRT-PCR (*n* = 5 to 6 per group). Data are presented as the mean ± SEM. ^∗^*P* < 0.05; ^∗∗^*P* < 0.01. All experiments were performed in triplicate.

Given that Tregs can modulate the Th immune response in the progression of experimental CS-induced lung disease [16], we examined the effects of DHI on the Tregs of CS-exposed mice. As shown in Figures 5(a)–5(d), CS exposure enhanced the number of Tregs in the HLN and spleen, which was decreased in a dose-dependent manner by DHI treatment at days 7 and 14. The expression levels of Foxp3 and TGF-*β*, the key transcription factor and effector cytokine for Treg cells, respectively, were also reduced following DHI treatment (Figures 5(e)–5(g)). These data indicated that DHI could regulate Treg cells at the CS-induced inflammation stage.

### 3.5. DHI Inhibited the Activation of STAT1 and STAT3 in CS-Exposed Lungs

**Figure 6:**
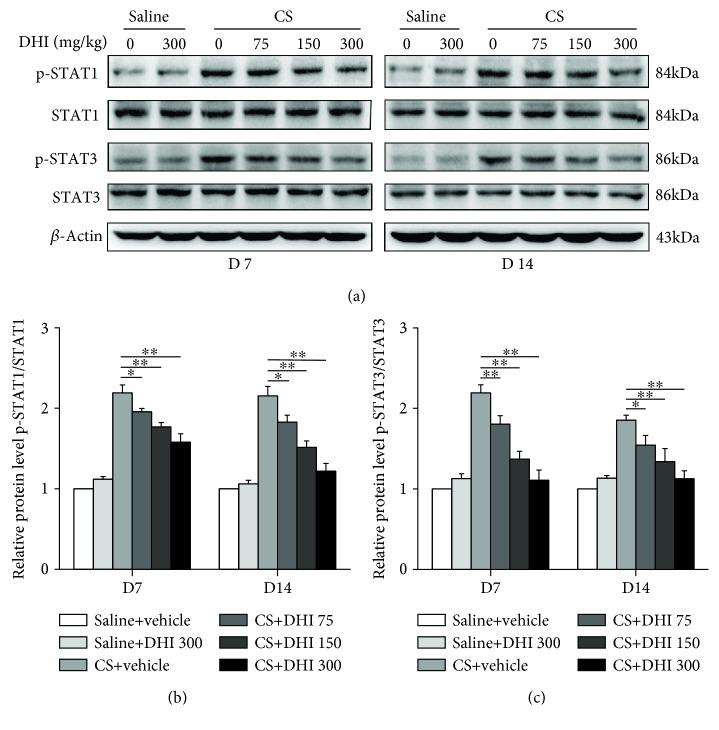
DHI inhibited the activation of STAT1 and STAT3 after CS injury. (a) Western blot analysis of STAT1, STAT3, and their phosphorylation in the lung tissues from different treated mice (*n* = 4 per group). (b, c) Quantification of phospho-STAT1 and phospho-STAT3 was shown as the ratio of phosphorylated to total protein, and the data were normalized to the saline control group. Data are presented as the mean ± SEM. ^∗^*P* < 0.05; ^∗∗^*P* < 0.01. All experiments were performed in triplicate.

To investigate the underlying mechanism of the effect of DHI on the immune response, we examined the effects of DHI on the proinflammatory transcription factors STAT1 and STAT3, which play crucial roles in the differentiation of Th cells [45]. As shown in Figure 6, DHI treatment reduced CS-induced phosphorylation of both STAT1 and STAT3 in mouse lung tissues in a dose-dependent manner. These results suggested that DHI could exert its immunoregulatory and anti-inflammatory effects by inhibiting the activation of STAT1 and STAT3.

## 4. Discussion

CS particles have long been known to cause silicosis, an incurable lung disease characterized by chronic inflammation and progressive fibrosis [1, 6]. Chronic silicosis resulting from long-term exposure to low concentrations of CS is the most common form that usually develops over 10 or more years [1]. However, it should be noted that acute silicosis, the most severe form, develops after short-term exposure to extremely high concentrations of CS, which probably results in silicosis deaths in young adults (ages 15 to 44 years) [1]. It is generally known that the accumulated CS cannot be removed from the lungs and induces persistent lung inflammation, which leads to lung injury, disability, and even death. It is widely accepted that the well-controlled inflammation will lead to alleviated fibrosis, since severe inflammation will enlarge the injury to the lung tissue [46]. Here, we used an experimental mouse model of CS exposure and selected the endpoints of 7 and 14 days to mimic the inflammation stage of silicosis. To determine the pathogenesis of CS-induced inflammation and identify potential new therapeutic strategies to treat this disease, we administered CS-exposed mice with DHI, a major constituent of Danshen that has multiple bioactivities. In the present study, we found that DHI treatment reduced CS-induced pulmonary inflammation. Its underlying mechanism appeared to involve the regulation of the Th immune response and inhibition of STAT1 and STAT3.

Inhalation of CS particles initiates a prominent pulmonary inflammatory response, which contributes to the development of CS-induced pulmonary disease. In response to CS, macrophages, which are the first defense in the innate immune response, recognize and take up inhaled CS particles. They then become activated to release inflammatory mediators, such as IL-1*β*, IL-6, and TNF-*α*. These inflammatory mediators induce persistent recruitment of inflammatory cells (macrophages, neutrophils, and lymphocytes) that lead to uncontrolled cycles of tissue damage [23]. Our results showed that DHI had a marked inhibitory effect on the CS-induced pulmonary inflammatory response. We found that DHI treatment reduced the overexpression of IL-1*β*, IL-6, and TNF-*α* in the lungs of CS-injured mice in a dose-dependent manner. DHI also significantly reduced the infiltration of inflammatory cells, particularly macrophages and lymphocytes. Given that B lymphocytes possess multiple regulatory effects on the immune system (e.g., producing massive amounts of proinflammatory cytokines and immunoregulatory cytokines; influencing T cell function) [47, 48], we investigated whether DHI administration could affect B cells and found that the accumulation of B cells in lung tissue of mice exposed to CS was effectively reduced by DHI.

Adaptive immunity is also involved in the development of CS-induced lung disease [42]. CD4+ T cells (e.g., Th1, Th2, Th17, and Treg cells) are key participants in the pathogenesis of CS-induced pulmonary disease [7, 8, 42]. Th1 cells usually function as inflammatory cells by secreting the proinflammatory cytokine IFN-*γ* to accelerate the clearance of accumulating CS particles, which may induce lung injury [46]. In this study, we found that DHI significantly suppressed the differentiation of Th1 cells and the production of the Th1-associated cytokine IFN-*γ* in CS-injured mice, which was consistent with a previous study that demonstrated that DHI strongly inhibited Th1-derived IFN-*γ* production in activated lymph node cells [41]. T-bet is an essential transcription factor for Th1 differentiation and IFN-*γ* production [49]. In addition, the IFN-*γ*-STAT1-T-bet-IFN-*γ* pathway has been reported to serve as a cogent amplification mechanism for Th1 differentiation in vitro [17]. Given our discovery that DHI treatment also suppressed T-bet expression and STAT1 activation in CS-injured mice, the combined effects of DHI treatment might account for the attenuated Th1 immune response.

Numerous studies suggest that the progression of CS-induced pulmonary disease is related to the disruption of the Th1/Th2 balance [4]. Th2 cells can inhibit the Th1 cell response [46]. We demonstrated that DHI treatment effectively decreased the production of the Th2-associated cytokines IL-4 and IL-13. However, the expression of GATA3, the Th2 master regulator, was not changed. Apart from Th2 cells, multiple cells can secrete IL-4 and IL-13, including mast cells, basophils, and IL-4- or IL-13-conditioned macrophages [50]. These cells may release IL-4 and IL-13 to limit the Th1 response. In turn, the attenuated Th1 response may contribute to a milder Th2 response to maintain immune homeostasis. These results may explain the regulatory effect of DHI on the Th2 response in CS-induced lung inflammation. However, further investigation is needed.

Th17 cells are the major producers of the IL-17 family of cytokines, which mediate the recruitment of inflammatory cells, improve B cell function, and elicit the release of other proinflammatory cytokines that jointly promote tissue injury in many inflammatory diseases [51]. Enhanced levels of Th17 cells and IL-17A in the lung tissue have been reported in CS-treated mice [5, 46]. Moreover, neutralization of IL-17A resulted in decreased CS-induced pulmonary inflammation [44]. In our study, we observed that DHI treatment abrogated the increase in the number of Th17 cells and IL-17A levels induced by CS. The induction of ROR-*γ*t, the master regulator for Th17 cell differentiation and IL-17A production [52], requires STAT3, which binds directly to the *Rorc* genes [22]. Many studies have shown that STAT3 plays a vital role in Th17 cell differentiation [53, 54]. IL-6 activation of STAT3 promotes the differentiation of Th17 cells by initiating the transcription of ROR-*γ*t [17, 22]. Our study showed that DHI could inhibit the activation of STAT3 in the lungs of CS-injured mice concomitant with a reduction in ROR-*γ*t expression. These findings may account for the inhibitory effect of DHI on the Th17 response.

Tregs (CD4+Foxp3+) are immune modulators with a crucial role in lung inflammation [55]. In the present study, we found that the number of Tregs in both the HLN and spleen was decreased by DHI treatment. There are several possible reasons for the decreased number of Tregs. First, a previous study showed that Tanshinone II-A (TNA) attenuated the Treg population in an inflammatory mouse model [56]. As an analog of TNA, DHI may exert a similar regulatory effect on the Treg population. Second, the anti-inflammatory effects of DHI prevented the activation of the inflammatory transcription factors STAT1 and STAT3 and weakened the T cell-associated immune response, thereby affecting the number of Tregs. Third, TGF-*β* can stimulate naive CD4+ T cells to differentiate into Foxp3+ Tregs [57]. We found that DHI treatment reduced TGF-*β* production, which could account for the DHI-mediated downregulation of the Treg cell population. Furthermore, it should be noted that although several natural products have demonstrated protective effects against CS-induced lung inflammation, their effects on Tregs were diametrically opposed [46, 58, 59]. In line with our results, dioscin and blue honeysuckle extract reduced the number of Tregs during the progression of CS-induced pulmonary inflammation [46, 58]. However, baicalin increased the number of Tregs in CS-exposed mice [59]. These findings suggested that the role of Tregs in CS-induced pulmonary inflammation is complicated, and the mechanism by which DHI modulates Tregs requires further investigation.

## 5. Conclusions

In summary, DHI treatment had protective effects against CS-induced pulmonary inflammation by regulating the Th immune response and suppressing STAT1 and STAT3 activation. Our findings not only shed new light on the mechanisms of the immunosuppressive and anti-inflammatory effects of DHI but also identify a potential new therapy for the prevention and control of CS-induced pulmonary disease.

## Figures and Tables

**Table 1:** Primer sequences for qRT-PCR.

*Mus musculus* gene name	Forward primer (5′-3′)	Reverse primer (5′-3′)
*Gapdh*	AGGTCGGTGTGAACGGATTTG	TGTAGACCATGTAGTTGAGGTCA
*T-bet*	AGCAAGGACGGCGAATGTT	GGGTGGACATATAAGCGGTTC
*Gata3*	CTCGGCCATTCGTACATGGAA	GGATACCTCTGCACCGTAGC
*Ror-γt*	ACGGCCCTGGTTCTCATCA	CCAAATTGTATTGCAGATGTTCCAC
*Foxp3*	CCCATCCCCAGGAGTCTTG	ACCATGACTAGGGGCACTGTA
*Tgf-β*	CTCCCGTGGCTTCTAGTGC	GCCTTAGTTTGGACAGGATCTG

## Data Availability

The data used to support the findings of this study are available from the corresponding author upon request.
